# Interactions among microorganisms functionally active for electron transfer and pollutant degradation in natural environments

**DOI:** 10.1016/j.eehl.2023.01.002

**Published:** 2023-01-27

**Authors:** Qixing Zhou, Ruixiang Li, Tian Li, Ruiren Zhou, Zelin Hou, Xiaolin Zhang

**Affiliations:** a MOE Key Laboratory of Pollution Processes and Environmental Criteria / Tianjin Key Laboratory of Environmental Remediation and Pollution Control, College of Environmental Science and Engineering, Nankai University, Tianjin 300350, China; b Department of Biological and Agricultural Engineering, Texas A&M University, TX 77843-2117, USA

**Keywords:** Electroactivity, Biodegradability, Microbial consortia, Biochemical interaction

## Abstract

Compared to single microbial strains, complex interactions between microbial consortia composed of various microorganisms have been shown to be effective in expanding ecological functions and accomplishing biological processes. Electroactive microorganisms (EMs) and degradable microorganisms (DMs) play vital roles in bioenergy production and the degradation of organic pollutants hazardous to human health. These microorganisms can strongly interact with other microorganisms and promote metabolic cooperation, thus facilitating electricity production and pollutant degradation. In this review, we describe several specific types of EMs and DMs based on their ability to adapt to different environments, and summarize the mechanism of EMs in extracellular electron transfer. The effects of interactions between EMs and DMs are evaluated in terms of electricity production and degradation efficiency. The principle of the enhancement in microbial consortia is also introduced, such as improved biomass, changed degradation pathways, and biocatalytic potentials, which are directly or indirectly conducive to human health.

## Highlights

• Environmental microorganisms are essential in solving pollution and energy crisis.
• The combination of various microorganisms facilitates the utilization of respective advantages.
• The characteristics of electroactive microorganisms and degradable microorganisms and their interactions are elucidated systematically.
• The enhancement of electricity production, hydrogen production, and pollutant removal capacity in microbial consortia was first analyzed.
• Call for research on functionalized microorganisms and microbial consortia.

## Graphical abstract

**Figure:**
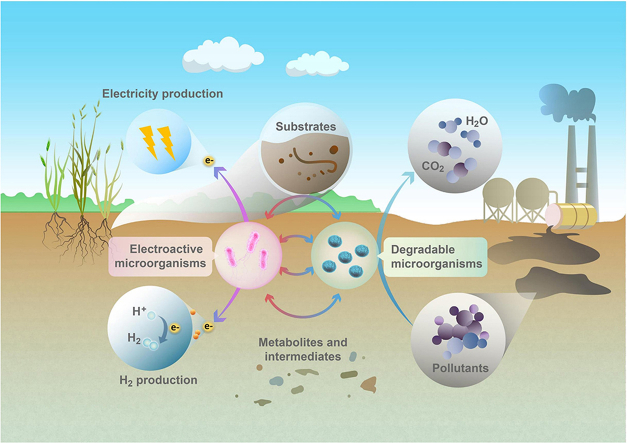

## 1 Introduction

Essential for human survival and development, microorganisms can grow on the earth’s surface or deep in the earth. They not only participate in the geological cycle of elements but also play an irreplaceable role in the formation and degradation of fossil fuels (natural gas, coals, and oils) and organic matter [1,2]. In the past decades, the ability of various microorganisms to biodegrade environmental pollutants and generate bioenergy has been extensively studied in response to widespread environmental pollution as well as energy shortages.

Given the expectation for green energy and environmental protection, electroactive microorganisms (EMs) and degradable microorganisms (DMs) are becoming two significant categories of microorganisms of interest. EMs are microorganisms with extracellular respiratory function; that is, microorganisms can completely oxidize intracellular organic matter to release electrons. The generated electrons can be passed through the intracellular respiratory chain to the extracellular electron acceptors to reduce and generate energy, sustaining the growth of microorganisms themselves [3]. This property of EMs allows them to perform different roles in different systems, including power generation, pollutant treatment, and hydrogen production [4]. Therefore, the presence of EMs can be indispensable in alleviating the energy crisis and pollution problems. However, the pollutants that EMs can utilize alone are limited and are easily degradable in structure. This means that EMs play the role of terminal or subterminal degradation in the degradation chain of complex pollutants, depending on their ability to extend the use of electron receptors [4]. The species of DMs are diverse, and here, we define them as all microorganisms capable of using organic matter and will therefore include some EMs. Based on current knowledge of degradation products and degradation capabilities of individual microorganisms, different organisms can be suggested to cooperate in the biodegradation of specific pollutants and the generation of value-added products. Here, we focus on interactions between EMs and DMs, and their energy-environmental impetus. These are bound to have important guiding significance for developing the bioelectricity industry and remedying contaminated soil and water and will further elevate the research level in the field of earth and environment.

To better understand the relationship between EMs and DMs, this paper comprehensively reviews the functions and characteristics of EMs and DMs. First, the types of EMs, the unique electron transfer mechanisms, and the available carbon sources are described. Next, DMs for different environmental survival are discussed. Then, the different functions corresponding to EMs and DMs and their interactions are presented. Finally, we discuss the challenges and application prospects of the current microbial communities composed of EMs and DMs to help researchers optimize the microbial communities and improve their performance.

## 2 EMs and their functional characteristics

In 1911, British botanist Potter discovered that the life activities of microorganisms metabolizing organics are accompanied by the release of electrical energy [5], which is due to the directional movement of extracellular electrons from EMs in the presence of exogenous electrodes. The metabolism of organics and the release of extracellular electrons catalyzed by EMs with their enzymes are the core reactions [6]. The enzymes in EMs, both intracellular and extracellular, replace traditional noble metal catalysts, reducing the activation energy of organics disintegration and accelerating the release of electrons. Moreover, it is important to note that in addition to the microorganisms that can produce extracellular electrons (namely electricigens or exoelectrogens), there are species that can use extracellular electrons released from other cells or electrodes (namely electrotrophs), which also participate in the extracellular electron transfer [7].

### 2.1 Introduction to the different EMs

EMs possess unique functions for the electron transfer of organics oxidation, and their new types are constantly being discovered. From the early electrical effects of fermented yeast and *Escherichia coli* to the later discovery of high-efficiency power generation capabilities of *Geobacter* spp. and *Shewanella* spp. to the current special mechanism of extracellular electron transfer in cable bacteria (belong to *Desulfobulbaceae*), the conclusion can be drawn that EMs are highly diverse and ubiquitous. Currently identified exoelectrogens are scattered in bacteria, fungi, and archaea. They survive on some of the energy generated by the oxidation of organics through the electron transport chain, while the other part of the energy is transported to electrodes along with the external electron flow preserved in the form of electric potential energy.

*Geobacter* is the most important exoelectrogens, and the *Geobacteraceae* family enriched on graphite or platinum electrodes can often generate continuous current in deep-sea sediment fuel cells [8]. More importantly, many genera of *Geobacter*, including *Geobacter metallireducens*, *G. sulferreducens*, *Desulfuromonas acetoxidans*, and *Geopsychrobacter electrodiphilus*, have also been shown to be able to use electrodes as the only electron acceptor to metabolize organics in systems without electron mediators. Completing the whole genome sequencing of *G. sulferreducens* has endowed it with the mission of a model exoelectrogens [9].

*Shewanella* is also rich in exoelectrogens, and *Shewanella putrefaciens* was proven to dissimilate reduced metals to produce electric current as early as 1999 [10]. Subsequent studies proved that *Shewanella oneidensis* DSP10 reached a very high power density (2 W/m^2^) in a microbial fuel cell (MFC) with a volume of 1.2 mL [11], and *S. oneidensis* MR-1 of various types of mutations have discernible power generation capabilities [12]. *Rhodoferax ferrireducens* is an iron redox microorganism, whose pure culture can also efficiently metabolize glucose to reduce electrodes [13]. The other exoelectrogens without the demand for electron mediators, *Desulfobulbulbaceae* (known as cable bacteria), can not only use electrodes as the only electron acceptor to oxidize S_0_ to SO_4_^2−^ and obtain energy but also grow with conductive multicellular filaments crossing the oxic-anoxic area (>1 cm in length) to achieve the long-distance electronic transmission [14].

With the assistance of electron mediators (exogenous or secreted by microorganisms), many bacteria can also transfer extracellular electrons to the electrode, such as *Escherichia coli*, *Pseudomonas,* and *Bacillus* [15,16]. For prokaryotic cells, *Saccharomycetaceae*, *Candida* sp. IR11, and *Blastobotrys adeninivorans* have been certificated to transfer electrons from the cell surface, and the secreted endogenous mediators, such as yeast extract, may accelerate this process [16,17]. Proving the universality of extracellular electron transfer, archaea that prefer extreme environments have also been found to be electrically active. Hyperthermophilic obligate anaerobes *Pyrococcus furiosus*, *Ferroglobus placidus*, and *Geoglobus Ahangari* can generate electricity at high temperatures (>80 °C), which extends the application scope of microbial electrochemical technology [18]. The microbes mentioned above are electricity-producing bacteria (exoelectrogens), and another type of EMs on the cathode (electrotrophs) can obtain electrons from the electrode to reduce the electron acceptor. For example, the pure culture of *Geobacter metallireducens* and *Alcaligenes faecalis* can reduce nitrate to nitrite on the cathode. *Desulfopila* and *Desulfovibrio* perform the electrotrophic reduction of sulfate, and *Klebsiella pneumoniae* can reduce the cathodic manganese oxide and oxygen [19,20].

EMs are highly differentiated in phylogeny primarily based on the diversity of extracellular electron transfer methods, which makes them lack reliable molecular biology technology (e.g., PCR primers, FISH probes) to identify a species and whether it can generate electricity. However, we can apply genetic engineering technology to modify existing bacteria. For example, the introduction of the Mtr pathway, which determines the specific extracellular electron transfer of *Shewanella monocytogenes* MR-1, or the phenazine-1-carboxylic acid (as an intermediate) synthesis pathway of *Pseudomonas aeruginosa* into *E. coli* can greatly enhance their power output capacity [21,22].

### 2.2 Extracellular electron transfer mechanisms and their development

Extracellular respiration is an electron transfer process evolved by dissimilar metal-reducing bacteria to transfer electrons released from oxidized intracellular organics to the outside of the cell under anaerobic conditions and reduce solid electron acceptors, thus obtaining the energy for their growth [7]. This unique electron transfer pathway makes it possible to transfer extracellular electrons along artificially placed electrodes and recover this part of the energy. In this way, the electron transfer mechanism from intracellular to extracellular and further to the electrode is a problem worthy of attention. It is well known that the metabolic activity of cells is carried out by a chain of electron transfer, and the transfer of electrons from the cell to the outside world requires the passage of a nonconductive inner membrane, a periplasm, and an outer membrane. Fortunately, there are also many proteins (enzymes) and other electron mediators (e.g., quinones) around the cell membrane and periplasmic space, which extend the electron transport chain outside the cell.

**Fig. 1:**
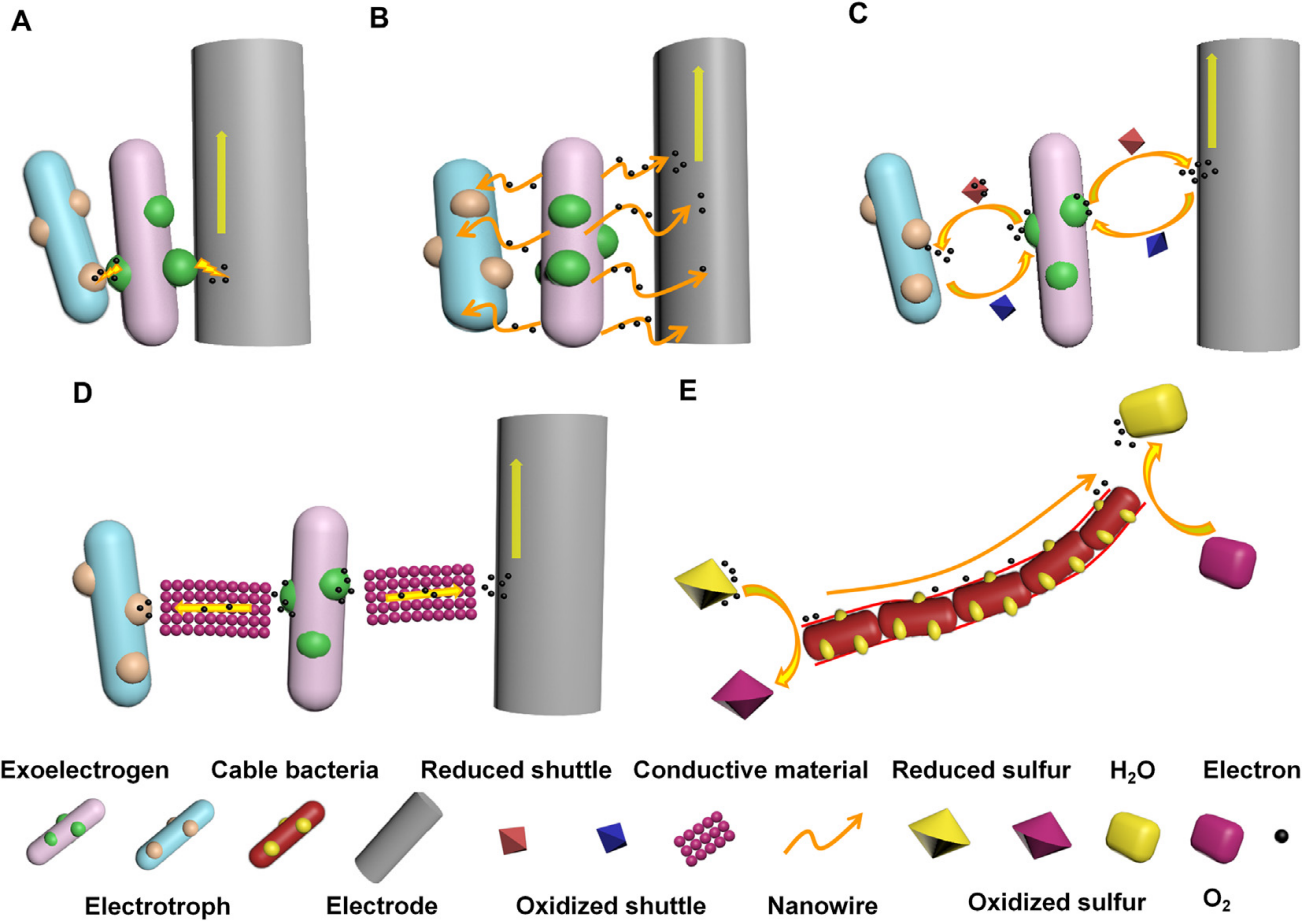
The electron transfer from exoelectrogen to the electrode or electrotroph shares the same mechanism. (A) The direct transfer by proteins associated with outer cell surfaces (e.g., *c*-type cytochrome) [23,26]; (B) the transfer by electrically conductive pili (nanowire) [[28], [29]]; (C) the indirect transfer via electron transfer shuttle [[15], [16]]; (D) the transfer mediated by conductive material [24,25]; (E) long-range transfer by the filament of cable bacteria via sulfur redox coupling [14].

The *c*-type cytochrome has been reported to be extremely important in electron transport across membranes. Previous studies have shown that three of the four proteins required for the Mtr pathway conferring extracellular electron transfer along a defined route of *S. oneidensis* MR-1 are *c*-type cytochromes [23]. The currently known mechanisms of interspecies electron transfer between EMs and other microorganisms rely mainly on direct interspecies electron transfer (DIET) and energy carriers in the form of hydrogen or formate to exchange metabolic electrons. This particular form of mutualism plays a key role in the methane degradation of organic matter in natural and engineered anaerobic ecosystems. In particular, the electron channels formed by magnetite (Fe_3_O_4_) can accelerate electron transport and lead to efficient cooperative catabolism [24,25]. Through whole genome sequencing analysis, *G. sulferreducens* owns about 100 genes encoding *c*-type cytochromes throughout the inner membrane, outer membrane, and periplasmic space, while *S. oneidensis* MR-1 has 37 similar genes [26]. Cells colonized on electrodes can rely on outer membrane cytochromes to transfer electrons directly to the conducting material (i.e., DIET) (Fig. 1A). Still, they account for only a small fraction of the power conducted by the biofilm. Some resourceful microorganisms slowly evolved, and conductive appendages such as conductive fimbria (nanowires) and vesicles gradually extended from the somatic cells, and even secreted electron shuttles to mediate the movement of electrons [27]. Previous Fe(III) reduction studies have discovered that special conductive pili of bacteria (called nanowires) can directly contact solid iron oxides to achieve electron transport [28]. *Geobacter* spp. has the property of forming a thick, highly conductive biofilm on the electrode, which uses pili to effectively transport electrons to the anode (Fig. 1B) [29]. Subsequent research has shown that the oxygen-enriched phototrophic cyanobacteria *Synechocystis* sp. PCC 6803 and *S. oneidensis* MR-1 can also produce conductive nanowires to transport electrons [30].

There is no consensus on the mechanism of conductivity of nanowires, but it was found that the conductivity of purified pilin is regulated by temperature, which is similar to that of nanostructured organometallics. Therefore, it is speculated that this metal-like conduction mode may be attributed to the π-π configuration between phenyl rings of phenylalanine or phenol rings of tyrosine contained in the pilin [31]. Additionally, the redox hopping between the cytochrome *OmcS* in the pili structure may facilitate electron transfer. With a closed bilayer membrane structure, vesicles (i.e., liposomes) are important transport carriers in the efflux process of microbial secretions. It may carry large molecular proteins (e.g., *c*-type cytochromes) and electron transport chains to the electrode. Electron mediators with reversible redox activity play an important role in long-distance electron transport without contact with conductive appendages (Fig. 1C). The electron mediator may have crucially influenced the initial discovery of electron transfer in EM yeast. Without other mediators, MFC enriched with *Saccharomycetaceae* and added with yeast extract achieved a higher power density (20–70 mW/m^2^) [15]. Later studies proved that *S. oneidensis* and *Bacillus subtilis* could synthesize riboflavin, and *Pseudomonas aeruginosa* could secrete phenazine substances (pyocyanin) to promote extracellular electron transfer [16]. In addition to the mediators secreted by the microorganisms themselves, synthetic electron shuttles or conductive materials (e.g., graphene, magnetite) added to the system can also accelerate the transfer of extracellular electrons to the electrodes (Fig. 1D). Inevitably, the possible toxicity of the mediator on microbial activity should also be taken into account.

Currently, the mechanism of extracellular electron transfer and human intervention is being deepened. The continuously elucidated electron transfer mechanism is an important theoretical basis for optimizing the structure of MFC/microbial electrolysis cell (MEC), modifying electrode materials, and improving electron transfer efficiency. In the short term, the greatly accelerated extracellular electron transfer will enable the application of microbial electrochemistry to break through the current limitations of either new energy production or remediation of contaminated media.

### 2.3 Full utilization of a variety of carbon sources by EMs

Recent studies have demonstrated that almost all microbially degradable organic matter can be utilized by EMs as fuel for microbial electrochemical systems (MFC/MEC), including simple substrates (e.g., carbohydrates and proteins) and complex organic mixtures or contaminants. The various substrate types in the anode may have an impact on the microbial community structure, resulting in differences in the electricity production of MFC and the hydrogen production efficiency of MEC.

Microbial electrochemical systems are used to remove a variety of contaminations, including hydrocarbons, pesticides, antibiotics, benzene, and organic wastewater. The systems involved contain pure/mixed culture, sole/complex substrates, and water/sediment/soil phases. The research of simple systems (with a sole substrate or pure bacteria) is convenient for revealing mechanisms of microbial reactions. The MFC fed with acetate, lactate, and glucose sharing the same concentration of 200 mg/L reached power densities of 48.4, 52.0, and 40.3 mW/m^2^, respectively. And the model exoelectrogens *Geobacter sulfurreducens* was found on all anodes of different systems, while a species of *Firmicutes* only appeared in glucose-fed MFC, which may be responsible for converting complex substrates into simple molecules [32]. Another study of MFC with different carbon sources of acetate, butyrate, and glucose also found that the power density of the system with glucose as the substrate was relatively low, and the enrichment of *Clostridium* and *Bacilli* was related to the glucose addition [33]. Eyiuche et al. [34] discovered that the type of substrate not only affected the power output of MFC, where the highest power generation occurred in the system fed with acetate, followed by peptone, starch, glucose, and wastewater but also caused differences in the structure of the microbial community. These results indicated that the conversion of macromolecular substrates (e.g., glucose) into electrical energy in MFC requires the participation of abundant microorganisms in addition to the exoelectrogens, accompanied by more energy loss. Moreover, the sole substrate of cysteine, ethanol, and cellulose could also induce differential evolution of microbial community, which can be divided into substrate-decomposing bacteria and metabolite-utilization bacteria (e.g., exoelectrogens). For complex systems, the types of substrates involved are usually diverse. For example, in a soil system contaminated by petroleum hydrocarbons, the organic substrates are not only rich in hydrocarbons, benzene, and other harmful substances but also humus, cellulose, and animal/plant residues preserved in the soil. Therefore, the participating functional bacteria are also versatile, with interactions between degrading bacteria, electrogenic bacteria, and element cycle bacteria.

For environmental remediation, MFC was originally applied to the treatment of organic wastewater, including biological, agricultural, and industrial wastewater, with the goal of reducing COD, TN, TP, and other indicators. The MFC wastewater treatment technology was first applied by professor Logan from the University of Pennsylvania in 2004. After continuous system optimization, including improvements in configuration, electrode materials, catalysts, and environmental conditions, it has gradually matured. Puig et al. [35] applied a single chamber air-cathode MFC to decontaminate the domestic sewage, achieving an 80% COD removal rate and a power density of 1.14 W/m^3^. In another oxic-anoxic biocathode coupled MFC, the TN removal rate of domestic sewage reached as high as 97.3% [36]. Farming wastewater usually contains a large amount of organics, suspended matter, nitrogen, and phosphorus and emits malodorous gas, which is discharged in a concentrated manner. Min et al. [37] used dual-chamber and single-chamber MFC to treat swine farm wastewater, respectively, showing that single-chamber MFC produced a high power density of 261 mW/m^2^ and the ammonia nitrogen removal rate reached 83%. Industrial wastewater often comes from food processing plants, paper mills, printing, and dyeing plants and has complex components with high biological toxicity.

Relying on its broad adaptability, the microorganism also performs well in MFCs contaminated by industrial wastewater. The success of MFC in wastewater treatment applications has stimulated the desire to apply it to soil and sediments. Soil MFC is currently widely used to remove a series of organic pollutants, including petroleum hydrocarbons, phenols, pesticides, antibiotics, esters, etc. Based on the abundance of soil microorganisms, the *Proteobacteria*, *Firmicutes*, *Bacteroidetes*, and *Actinobacteria* are generally the dominant bacteria at the phylum level in soil MFC, and many of them are involved in electricity production and degradation. With the degradation of organic pollutants and the production of biocurrent, the selective enrichment of specific microbial species has occurred. For example, in the soil MFC for repairing petroleum hydrocarbons and the herbicide isoproturon, the *Clostridia* in the *Firmicutes* was significantly enriched under the stimulation of biocurrent [38].

## 3 DMs and their functional characteristics

In the last decades, more attention has been paid to DMs due to their application in the remediation of contaminated soil and water [2,39]. DMs produce enzymes or trigger redox reactions during biodegradation, which is also determined by the activity of DMs [39,40]. Many factors can affect the activity and thus impact biodegradation efficiency. Although some extreme environmental conditions initially lead DMs to an unfavorable direction, there is also a survival of the fittest after generations of selection. Nowadays, pollutants in mild environments can be efficiently degraded by microorganisms. Still, some DMs present in extreme conditions also play a key role in the remediation of contaminated environments [2].

### 3.1 Acidophilic and basophilic microorganisms

The degradation efficiency of microorganisms is related to the variation in pH. However, some DMs show high activity even in extremely acidic or alkaline environments. For example, acidophilic iron-oxidizing microorganisms play an important role in the recovery of copper and zinc from low pH ores. Some acidophilic iron-oxidizing microorganisms, such as *Acidithiobacillus ferrooxidans*, *Leptospirillum ferriphilum,* and *Leptospirillum ferrooxidans*, are widely used in Fenton biotreatment technology. Acidophilic microorganisms helped to adjust the pH of the Fenton process from extremely acidic to near neutral and save time for pre-neutralization, thus greatly improving the degradation efficiency [41]. Wang et al. [42] combined Fenton with DMs to achieve effective degradation of toxic organic pollutants without any pre-neutralization. Similarly, basophilic microorganisms are widely present in the degradation of organic pollutants, especially in soil and sludge. Basophilic microorganisms secret catalase and urease to improve soil health situations and promote the degradation of organic matter by breaking chemical bonds [43]. For example, *Mycobacterium* sp., a common DM of polycyclic aromatic hydrocarbons (PAHs), can be adapted to alkaline soil for pyrene degradation [44]. Recent studies have shown that alkaline pretreatment is often used to accelerate the hydrolysis step of sludge and improve digestibility. Through the combination of alkalinity and ultrasound, the sludge solubility is enhanced, and more low molecular weight substances are produced. Thus, extreme environments are not favorable for pollutant degradation, but some specific microorganisms survive from extreme environments and act as carbon sources to achieve pollutant degradation. If some methods are used to selectively enrich DMs, effective degradation of organic pollutants can be achieved in extreme environments.

### 3.2 Anaerobic and aerobic microorganisms

**Fig. 2:**
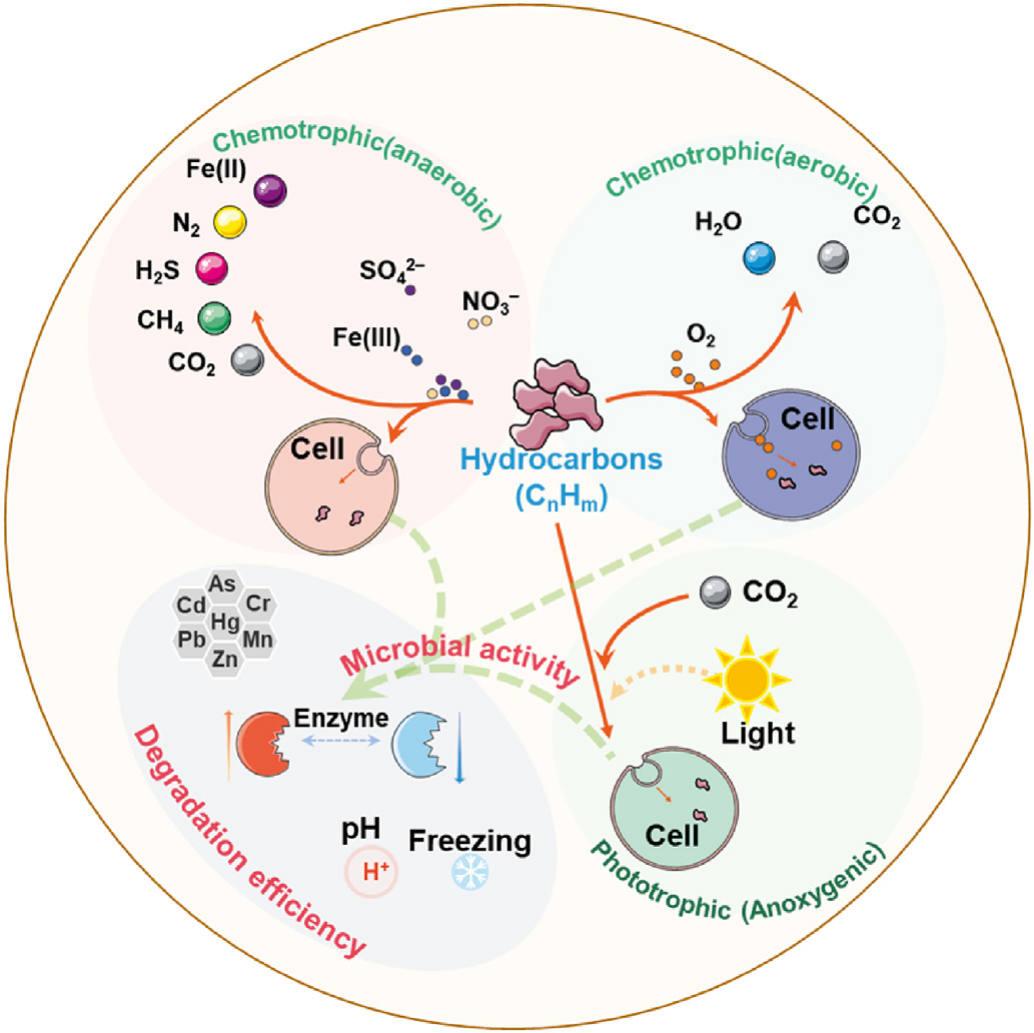
The possibility of microbial utilization of hydrocarbons. In chemical nutrient reactions, hydrocarbons are oxidized to maintain energy (catabolism), and another part is taken up by cells. The black label represents the name; The green label represents the degradation type; The red label represents the metabolic process affected by environmental factors; The solid line represents the direct process, and the dotted line represents the indirect process.

Anaerobic environments are usually associated with more anaerobic reactions with some anaerobic microorganisms. Methanogens, one of the most important microorganisms in anaerobic digestion, have been found in many anaerobic environments [45]. Anaerobic digestion is a common technique in this process, and anaerobic bacteria (including *Geobacter*, *Methanosaeta*, *Methanobacterium*, *Syntrophomonas*, and *Syntrophorhabdus*) play an important role in improving the degradation efficiency of organics and methane yield [46,47]. Meanwhile, some studies found that granular activated carbon could improve anaerobic biodegradation due to its high adsorption capacity of organic matter, and that the addition of hydrochar from different feedstocks could promote the growth of synthetic bacteria and direct interspecies electron transfer processes. Thus, the low conversion efficiency in anaerobic digestion was optimized due to the inherent complexity of hydrothermal liquefaction wastewater [46]. Anaerobic biodegradation also plays an important role in the removal of petroleum hydrocarbons. Nitrate, ferrous, manganese, or sulfate ions have been reported to be electron acceptors for the anaerobic degradation of petroleum hydrocarbons under anaerobic conditions (Fig. 2) [48]. Aromatic compounds are first oxidized to phenols or organic acids, then converted to long-chain volatile fatty acids, and finally metabolized to CH_4_ and CO_2_ [49]. In addition, extreme environmental media, such as desert soil, Antarctica’s underground ice, peatlands, and ocean margin, have been found to be subject to anaerobic biodegradation processes [50]. The understanding of aerobic degradation is more detailed as compared with anaerobic degradation. The presence of oxygen is also essential for the degradation of certain organic substances, since some microorganisms are aerobic. Liu et al. [51] conducted an indoor experiment on naphthalene-degrading microorganism isolated from contaminated soil in a graphite mine and showed that the naphthalene-degrading microorganism in contact with the graphitic materials promoted the oxidation reaction and formed oxidized sheets exfoliated, indicating the ability of the strain to oxidize graphite under aerobic conditions. Aerobic microorganisms have also been used in other applications. For effective waste recycling, plant residues and animal manure are often mixed for composting, and aerobic microorganisms spontaneously oxidize, decompose, and exothermically convert these materials into usable organic substrates, including microorganisms and fungi [52,53].

### 3.3 Thermophilic and psychrophilic microorganisms

During the long-term accumulation of plant remains, anoxia in moist or surface water environments slows down microbial metabolic activity, leading to the slow decomposition of organic matter and the formation of peatlands that contribute to the global C cycle. Wilson et al. [54] reported that CH_4_ production from surface peat increased significantly with increasing temperature, and the highest yield of CH_4_ was obtained from 20 to 30 cm peat at 14–16 °C. In addition, cellulose is the main component of polymers in plant remains, as it relies mainly on cellulases to convert insoluble cellulose substrates into soluble sugars (mainly cellulose and glucose). In particular, some cellulolytic microbes can work in an extremely high-temperature environment, including *Desulfurococcus fermentans* that act alone at an optimum temperature of 81 °C [55], or a consortium of three hyperthermophilic archaea (*Ignisphaera aggregans* DSM 17230, *Pyrobaculum islandicum* DSM 4184, and *Thermofilum pendens* Hrk) that can deconstruct lignocellulosic at 90 °C [56]. Thermophilic bacteria also appeared in the composting process, as reported by Zhu et al. [57]. In cattle manure composting, thermal pretreatment accelerated the rise in composting temperature, increased the degree of humus, and promoted the degradation and decay of lignocellulose by stimulating the vital metabolism of thermophilic bacteria. Conversely, certain bacteria seem to prefer low temperatures. Oil spills inevitably contaminate marine ecosystems with polycyclic aromatic hydrocarbons (PAHs), polychlorinated biphenyls (PCBs), and phenols during oil extraction and transportation [58]. In Antarctica, the extremely low temperatures are not inconducive to biodegradation. However, a hydrocarbonoclastic bacterium, *Oleispira antarctica* RB-8, isolated from Antarctic coastal marine environments, was shown to have a dominant role in petroleum degradation in cold and deep-sea environments, and its ability for alkane degradation, siderophore production, and micronutrients scavenging were also discussed [59]. Similarly, Antarctica is a reservoir of metabolically active microbial cells and organic carbon, with unique low temperature and pressure conditions conducive to methanogenic archaea degrading organic matter and producing methane hydrate [50]. In combination with sequencing techniques, it was shown that the genome of the psychrotolerant bacterium *Rhodococcus* sp. strain AQ5-07, which used phenol as its sole carbon and energy source, was involved in the complete enzyme system required for phenol and catechol degradation [60]. These works provide references and information for the genetic candidates responsible for the microbial degradation of organic matter in cold environments by native Antarctic bacteria.

### 3.4 Degradable bacteria under nutrient-deficient conditions

Soil nitrogen availability regulates soil microbial communities and organic matter decomposition and formation and has an important impact on the global carbon cycle. Compared to bacteria, fungi have lower nutrient requirements and lower metabolic activity, thus playing a dominant role in the decomposition of SOM. Therefore, the addition of nitrogen can reduce the ratio of fungi to bacteria. Zhou et al. [61] investigated the response of soil microbial community to nitrogen addition and found that changes in microbial species may strengthen or reduce the impact of microbial biomass change at the ecosystem level, highlighting the critical role of microbial community composition in soil ecosystem function under nitrogen deposition scenarios. Changes in microbial degradation efficiency by nitrogen accumulation are also present in the aqueous environment. Marine ecosystems are known to be rich in organic components due to their high species diversity. In addition, seawater exchanges material with the beach through waves or tides, resulting in a large amount of organic matter remaining in the sand pores. It is thought that many species of microorganisms are present in the pore spaces, and therefore, the chemical composition of the pore water has changed before being reintroduced into the ocean. However, Ahrens et al. [62] reported the effects of temperature-dependent seasonal cycles on organic matter and nitrate availability. For example, the beach sands of Spiekeroog were a sink for nitrogen in winter and early spring and served as a source of nitrogen for seawater in summer, which ultimately altered microbial rates and degradation pathways. The nutrient utilization efficiency of microorganisms also limits the degradation of organic matter. The biomass of soil microorganisms is a sensitive indicator of microbial changes in contaminated soils with increasing concentrations of heavy metals. Short-term or long-term exposure to toxic metals will lead to a decrease in soil microbial diversity and activity, which is detrimental to the degradation of organic pollutants. Zhang et al. [63] reported that the bacterial diversity of restored soils by reducing metal concentrations was higher than that of lead and cadmium contaminated soil. This phenomenon is mainly attributed to the inhibition of microbial respiration and metabolism [64]. In addition, genes encoding resistance to heavy metals were more prevalent under the long-term selection pressure of heavy metals [65,66]. For example, *Cupriavidus* sp. MTS-7, an acid-tolerant, diazotrophic P-solubilizing, and heavy metal-resistant bacterium, has been identified for its potential to degrade polyaromatic hydrocarbons in chronically mixed contaminated soils [44]. Similarly, fungi exhibited similar metal resistance compared to bacteria. Jiang et al. [65] found that *Debaryomyces* JS4 utilized phenol as the sole source of carbon and energy and could facilitate the bioremediation of phenol-contaminated industrial wastewater under heavy metal conditions.

## 4 Promotion of EMs performance by DMs

Microorganisms can not only interact with plants and animals but also promote their metabolism by interacting with other microorganisms around them. Interaction of microorganisms can be accomplished between similar or diverse species across genera and families. According to the different effects on each other, the interaction of microorganisms can be classified as syntrophic, neutral, predatory, and competitive [67]. EM refers to a type of microorganism that can exchange electrons with extracellular electron donors or acceptors through extracellular electron transport. Generally, in bioelectrochemical systems (BESs), EMs can act as biocatalysts, oxidize organic and inorganic substrates at the anode, or reduce substrates at the cathode. To generate electricity, the transfer of these electrons from inside the cells to the anode in anoxic conditions is required. The most widely studied EMs include *Geobacter* spp*.* [68], *Shewanella* spp*.* [30], *R. ferrireducens* [69], *P. aeruginosa* [70], and *B. subtilis* [71]. Although pure EMs can generate electricity and valuable products (e.g., H_2_, CH_4_, and H_2_O_2_) through their metabolism, such product is relatively low and difficult to collect [9]. In contrast, using a mixed culture is more practical and shows a broader range of organic compounds [72]. Microorganisms working together have been proven effective in improving the performance of EMs because of their higher adaptability to environmental changes. DMs are capable of decomposing macromolecular substances into small molecules. The enzymes, electrons, or intermediate products produced during the decomposition process can promote some properties of EMs.

### 4.1 Promotion of EMs electricity production by DMs

Generally, EMs can convert the chemical bond energy of organic substrates into electric energy without extra intermediate stages by utilizing electron transferring capability. Although EMs have been proven to produce satisfactory power generation, most conclusions were obtained in the laboratory. The substrates used for electricity generation are often simple organic substances (such as glucose, sodium, and acetate). In the actual environment, pure EM is not considered an effective inoculum because the substrates often consist of many complex substances, some of which even have toxic effects on microorganisms [59]. Therefore, using pure microorganisms cannot meet the demand for electricity generation for its low power generation. To enable BESs to meet the requirements of practical applications, researchers have tried numerous methods. Although exogenous mediators can be effective in increasing the power production of BES, environmental factors, the impact of the mediators, and costs must be taken into account. Recently, many research initiatives have focused on the interactions of different microbial communities. A large number of findings have shown that power generation through interactions is usually higher than that of pure microorganisms [73,74]. The EMs mixed with DMs are more tolerant to complex substrates and extreme environmental conditions and suitable for application in nature (e.g., wastewater, soil, and sediments). Table 1 shows some practical examples of using mixed cultures to enhance power generation.

**Table 1:** The application of microbial consortia in enhancing power generation.

Electroactive microorganisms	Degradable microorganisms	Current density/power density	Effect	Ref.
*Pseudomonas aeruginosa* PA14	*Enterobacter aerogenes*	46.53 μA/cm^2^	Increased by at least 14 times	[75]
*Pseudomonas aeruginosa*	*Enterobacter aerogenes*	212.00 μA/cm^2^	12 times higher	[76]
*Pseudomonas aeruginosa*	*Klebsiella variicola*	11.80 W/m^3^	3 times higher	[77]
*Pseudomonas aeruginosa*	*Escherichia coli*	209.30 μW/m^2^	7 times higher	[78]
*Lipomyces starkeyi*	*Klebsiella pneumonia*	12.87 W/m^3^	3 and 6 times higher	[79]
*Bacillus cereus*	Microorganisms in anaerobic sludge	4.83 W/m^3^	2.6 times higher	[80]
*Shewanella oneidensis*	*Trametes versicolor*	0.78 W/m^3^	Increased by 40%	[81]
*Shewanella oneidensis*	*Saccharomyces cerevisiae*	123.40 mW/m^2^	1.7 times higher	[82]
*Shewanella oneidensis*	*Pseudomonas putida*	2.00 μA/cm^2^	2.67 times higher	[83]
*Shewanella oneidensis*	*Escherichia coli*	3.00 μA/cm^2^	3 times higher	[84]
*Geobacter sulfurreducens*	*Achromobacter insolitus*	1.00 A/m^2^	Stimulated the power generation	[85]
*Bacillus subtilis* *Shewanella oneidensis*	*Escherichia coli*	241.50 mW/m^2^	5 times higher	[86]

**Fig. 3:**
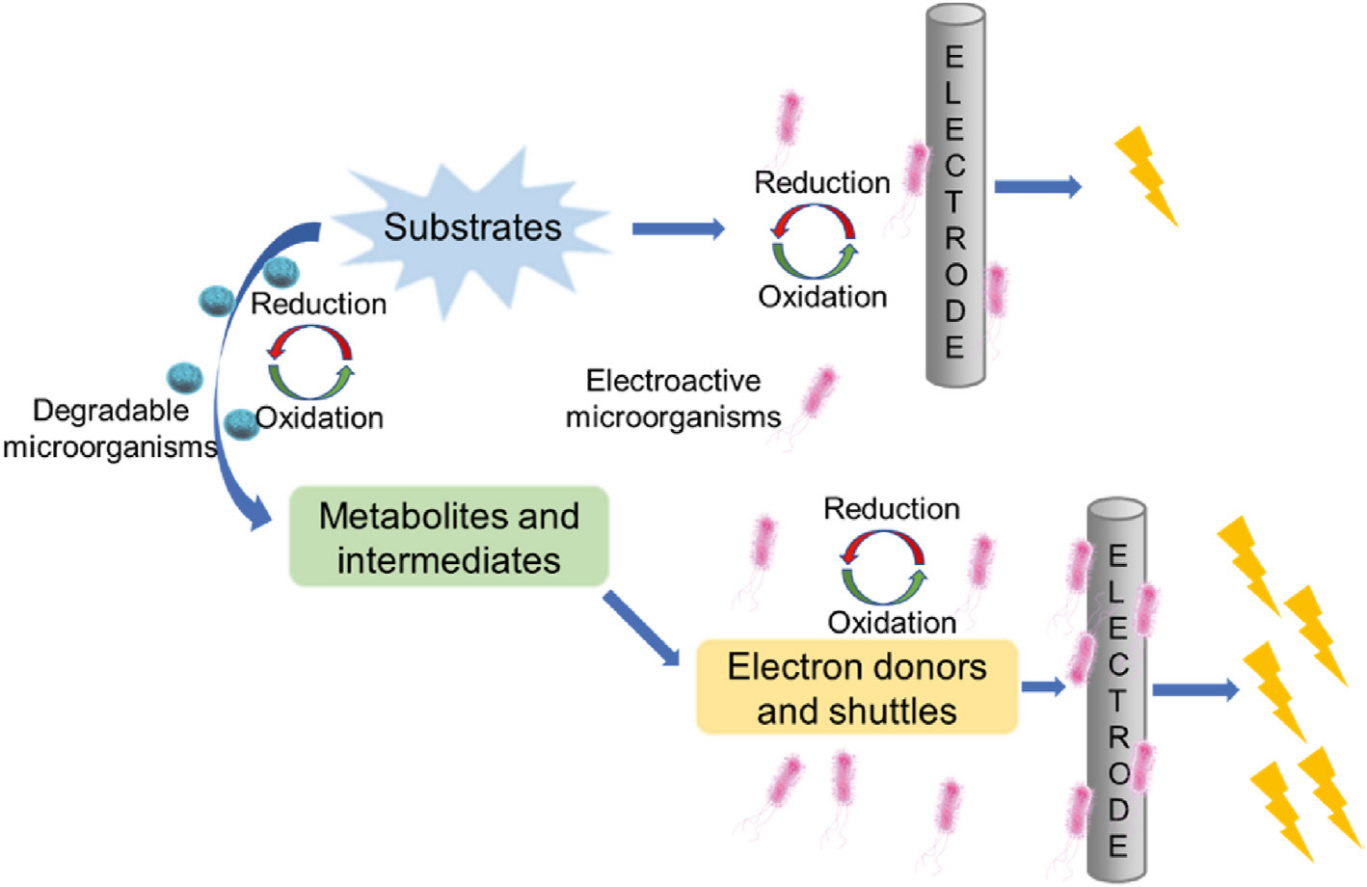
The schematic diagram of the mutualistic relationship between electroactive microorganisms and degradable microorganisms to generate electricity.

The addition of various DMs has different ways and mechanisms to improve the power generation capacity of EMs. As shown in Fig. 3, the main mechanism is that DMs convert the substances into other substances that can be used by EMs to produce more electron shuttling mediators and increase power production. For example, Venkataraman et al. [75] constructed a microbial system cocultured by *Pseudomonas aeruginosa* PA14 and *Enterobacter aerogenes* in a BES with glucose as the initial substrate under microaerobic conditions, and reported that the current density by a coculture of *P. aeruginosa* and *E. aerogenes* increased by at least 14 times compared to the current density by either of these two bacteria alone. They then identified the benefits of each microorganism in this symbiotic relationship, and believed that DMs *E. aerogenes* converted the glucose into 2,3-butanediol, which was subsequently consumed by *P. aeruginosa*, because the production of pyocyanin by *P. aeruginosa* was enhanced with the increase of 2,3-butanediol. It is worth noting that pyocyanin acts as an electron shuttling mediator, which could facilitate efficient electron transfer and thereby enhances power generation. Schmitz and Rosenbaum [76] used these two microorganisms to construct a coculture system and operate in the fed-batch mode BESs, where *Pseudomonas aeruginosa* and *Enterobacter aerogenes* formed a synergistic coculture and strongly enabled the BES to obtain very high current densities. In their experiment, the coculture of *P. aeruginosa* and *Klebsiella variicola* achieved approximately 3-fold power generation compared with the pure culture. They also attributed the improvement to the production of the fermentative metabolite (1, 3-propanediol) by *K. variicola*, which subsequently stimulated the production of pyocyanin by *P. aeruginosa*, and thereby the power generation of coculture MFC increased.

In BESs, biofilms on the anode surface play vital roles in power generation. Therefore, the thickness of the biofilm has a significant impact on power generation by affecting the mass transfer and nutrient diffusion. Many kinds of microorganisms on the biofilm can be divided into nonelectrogenic (especially methanogenic) and electrogenic microorganisms. If nonelectrogenic microorganisms dominate the biofilm, the electron transfer would be significantly hindered, reducing power generation [76]. Thus, the other mechanism to promote the power generation of EMs is to promote the formation of electroactive biofilms (EAB) and inhibit other microorganisms that can consume the electron. Islam et al. [80] proved this mechanism in a microbial community composed of *Bacillus cereus* and microorganisms in anaerobic sludge (AS). Compared with the MFC inoculated with AS, the mutualistic interactions between *B. cereus* and AS improved the power generation from 1.82 W/m^3^ to 4.83 W/m^3^. As commonly known, the microorganisms in AS were mainly composed of methanogens, which inhibited the growth of EMs, and consumed a lot of electron loss. The addition of *B. cereus* could produce large amounts of fatty acids capable of suppressing methanogenesis, promoting EMs to form EAB on the electrode surface. The other reason for the improvement of power generation might be that *B. cereus* was able to produce enzymes (e.g., lipase, protease, amylase, and cellulase), which can efficiently hydrolyze complex substrates into simple substrates that can be utilized by other EMs.

In addition to constructing microbial consortia with EMs and degradable bacteria, some researchers have constructed microbial consortia by using EMs and fungi to improve electricity production capacity for their faster growth, high tolerance, and wide range of substrates [17]. de Dios et al. [81] constructed the coculture system with the fungus *Trametes versicolor* and bacteria *S. oneidensis*, and reported that this MFC system provided a maximum volumetric power density of 0.78 W/m^3^, almost three times higher than that of pure EMs. The enhancement was mainly because that *T. versicolor* and *S. oneidensis* formed a network so that the bacterium would move onto the surface of the hyphae and transport the electrons to the anode. In addition, *T. versicolor* could produce a variety of oxidative enzymes, providing an oxidoreductase mechanism between the donor and the acceptor. Instead of forming a network to allow better electron transfer, some fungal-bacterial systems increase electricity production capacity by providing carbon sources for EMs. Lin et al. [82] used glucose-fed MFC inoculated with *Saccharomyces cerevisiae* and *S. oneidensis* and improved the maximum power density from 71.52 mW/m^2^ to 123.4 mW/m^2^. They attributed this improvement to overcoming the limitation of carbon sources available for exogenous electrons.

### 4.2 Promotion of EMs hydrogen production performance by DMs

Recovering energy from waste resources (e.g., wastewater, waste gas, and contaminated soil) can effectively alleviate the issue of energy shortage. With the development of biotechnology, more and more economical methods have been applied to recover valuable products from waste effectively. In some cases, EMs can accomplish simultaneous pollutant treatment and resource recovery via utilizing a range of organic substrates and interactions with electrodes. However, some problems exist in the application of EMs to convert waste into valuable products. For example, the composition of actual pollutants is very complex, while EMs that have been proven effective in the laboratory are often targeted at a single substrate. In comparison, combining EMs with other DMs to treat waste can achieve satisfactory results [87].

Hydrogen (H_2_) is universally accepted as an environmentally safe, renewable energy resource. The microbial consortia for H_2_ production can not only improve the output of H_2_ but also save more energy input than conventional methods. Bioenzymes are organic substances with catalytic effects produced by living microorganisms, which play an important role in H_2_ production. Therefore, H_2_ production can be promoted by increasing the production of different enzymes. Wang et al. [88] constructed a microbial consortium, consisting of *Bacillus cereus* and *Brevundimonas naejangsanensis* to improve H_2_ production. In the case of using starch as a substrate, this consortium produced 1698.5 mL/L H_2_, which was 2.4 and 1.7 times that of the pure cultures of *Bacillus cereus* and *Brevundimonas naejangsanensis*, respectively. They attributed the increase in H_2_ production mainly to mutual interactions. The α-amylase and glucoamylase that were functionally complementary in starch hydrolysis were secreted in large quantities. Additionally, *Bacillus cereus* converted the complex compound starch into lactate and electron donor, then *Brevundimonas naejangsanensis* used the simple substrates to promote the generation of H_2_. Meanwhile, in return, *Brevundimonas naejangsanensis* produced the electron shuttle to *Bacillus cereus* to enhance H_2_ production. The increase in H_2_ production can also be achieved by producing more extracellular electron shuttles in consortia. Zhang et al. [89] added reduced extracellular electron shuttle anthrahydroquinone-2, 6-disulfonate (AH_2_QDS) in the *Clostridium beijerinckii* and *G. metallireducens* coculture system to enhanced the H_2_ production. Compared with the pure culture of *C. beijerinckii*, the maximum H_2_ production increases by 52.3%. They reported that the coculture of *C. beijerinckii* and *G. metallireducens* improved xylose utilization and increased the amount of the extracellular electron shuttles used for biohydrogen production. This is because this consortium could regenerate the AH_2_QDS *in situ* and reduce the acetate accumulation during xylose fermentation.

## 5 Interactions between DMs and EMs in degradation performance

**Fig. 4:**
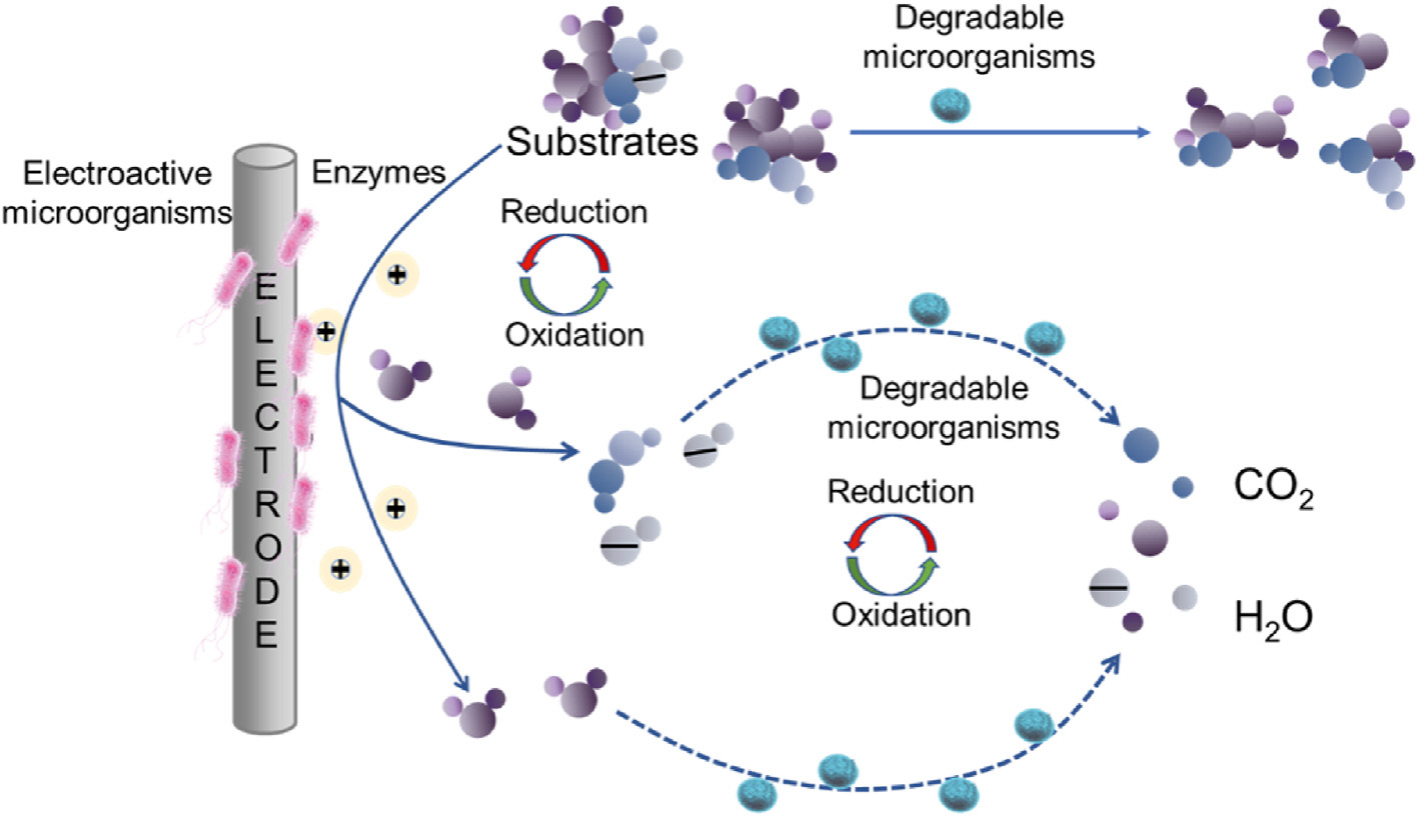
The schematic diagram of the mutualistic relationship between electroactive microorganisms and degradable microorganisms to degrade different compounds.

Although specific effects of single DMs on the degradation or removal of pollutants have been reported, there are still problems in practical applications. It has been shown that the bioremediation process of microorganisms can be enhanced by synergistic effects in the consortium (Fig. 4). In turn, the synergistic effect may be attributed to the enhanced metabolic activity resulting from the positive association between the interacting microorganisms. Meanwhile, an increasing number of researchers have reported that the application of microbial consortia composed of EMs and DMs can significantly improve degradation performance due to their multiple inherent functions and high adaptability.

### 5.1 Promotion of DMs degradation performance by EMs

Degradable bacteria can degrade or convert pollutants into less toxic substances through their metabolic activities. However, the degradation efficiency of pollutants using single DMs is very low and easily affected. In addition, the toxicity of pollutants may have a great influence on the survival of pure DMs. In contrast, they can survive and grow if the primary toxic substance is converted into less or negligible toxic forms by other EMs in the consortium. As shown in Table 2, in recent years, various consortia have been constructed to improve the degradation efficiency of different pollutants.

**Table 2:** Enhancement effect of microbial consortia in improving pollutant removal efficiency.

Pollutant	Microbial consortia	Effect	Ref.
Phenanthrene	*Escherichia coli* HY1 *and P. aeruginosa* PH2	1.6 times higher	[90]
Anthracene, phenanthrene, and pyrene	Electrogenic bacteria (dominated by *Geobacter*) and degradation bacteria	Increased from 22%, 19%, and 13% to 57%, 45%, and 29%	[91]
Chrysene	*Rhodococcus* sp., *Bacillus* sp., and *Burkholderia* sp.	Increased from 24% to 96%	[92]
O-xylene	*Shewanella oneidensis* MR-1 and mixed cultures	Increased from 54% to 73%	[93]
Phenol	Mixed culture (mainly includes *Geobacter*) and *Acidovorax*	Reached 80%	[94]
Congo red	*Pseudomonas putida* and *Shewanella oneidensis*	Increased from close to 0% to around 95%	[83]
Acid orange 7	Mixed inoculum of anaerobic sludge and *Shewanella oneidensis*	Reached 98%	[95]
P-chloronitrobenzene	*Pseudomonas fluorescens* and industrial sludge	Reached 100%	[96]
Glycerol	*Shewanella oneidensis* MR-1 and *Klebsiella pneumonae* J2B	Increased from 3% to 97%	[97]
Cellulose	*Clostridium cellulolyticum* and *Geobacter sulfurreducens*	Increase from 42% to 64%	[73]

The leaks and accidental spills of petroleum hydrocarbons and their derivatives during the exploration, production, transport, and storage processes have caused massive environmental pollution. The major hazardous chemicals in petroleum that seriously threaten human health and ecosystem safety [98] include BTEX (benzene, toluene, ethylbenzene, and xylenes), and PAHs. Consequently, it is urgent to find highly effective, widely applicable, and economical remediation technologies to deal with petroleum pollution. Compared with physical and chemical remediation, bioremediation using DMs has been shown to be effective and environmentally friendly. Petroleum hydrocarbons can be degraded biologically through different biochemical pathways [94]. However, bioremediation performance is affected by many factors, such as the abundance of electron acceptors, bacterial competition, and the toxicity of petroleum pollutants. Recently, the above limitations have been overcome by adding EMs into DMs to construct microbial consortia. Phenanthrene is a typical PAH, and can be treated well with microbial consortia. Jia et al. [90] isolated a phenanthrene DM, *Pseudomonas aeruginosa* PH1 from oil-contaminated soil, and constructed the co-culture system with *Escherichia coli* HY1 and *P. aeruginosa* PH2 to treat phenanthrene. Phenanthrene removal rates by the coculture consortium increased by 160% more than those by the pure *P. aeruginosa* PH1. They then examined the degradation pathway and found that the microbial consortium first oxidized phenanthrene to 9,10-dihydroxy phenanthrene and 1,2-dihydroxy phenanthrene and metabolized these intermediates via catechol to carbon dioxide and water. These microorganisms in the consortium played different roles, as *E. coli* HY1 functioned more in the initial oxidation process, while *P. aeruginosa* PH2 was primarily responsible for the ring cleavage. O-xylene, as a petroleum product, also can be removed by microbial consortia. You et al. [93] added *S. oneidensis* MR-1 to a microbial community in a BES to enhance the degradation of a recalcitrant organic compound, o-xylene. The experimental results revealed that the o-xylene removal efficiency increased from 54% to 73% after adding *S. oneidensis* MR-1. They attributed the improvement to the enhancement of the biofilm in the bioanode and the production of the nanowires between the bacteria. The addition of *S. oneidensis* MR-1 enabled the enrichment of *Hydrogenophaga* sp., *Chryseobacterium* sp., and *Sedimentibacter* sp., which could degrade oxylene, thus increasing the o-xylene degradation efficiency. What’s more, the OmcA-MtrCAB protein may be involved in direct electron transfer on *S. oneidensis* MR-1 containing biofilms. In addition to mixed hydrocarbon contaminants, pure chemicals have been tested in lab-scale studies. Shen et al. [94] used acetate as the substrate for the microbial consortium to remove phenols and achieved 79% removal efficiency. In this consortium, different kinds of microorganisms functioned differently. *Geobacter, Anaerovorax*, and *Devosia* can transport electrons to electrodes via outer-surface, *c*-type cytochromes, conductive pili, and secreted electron shuttles. Therefore, they are considered typical EMs. The DMs, including *Acidovorax*, *Thauera*, *Comamonas*, and *Sedimentibacter*, were found to be capable of degrading phenolic and aromatic compounds in biological treatment processes. Acetate promoted the growth of functional species responsible for phenol degradation and power generation.

In terms of wastewater eutrophication, the main task is to remove the excess elements, especially nitrogen. Normally, traditional physical and chemical methods to remove nitrogen have the limitation of producing secondary pollution and consuming a lot of energy. Through the metabolism of different microorganisms, nitrogen in water can be efficiently and rapidly removed, and even the purpose of recycling can be achieved. Rahimi et al. [99] cocultured *B. subtilis* with the microbial communities present in wastewater with high concentrations of nitrate or ammonium and added 1% glucose to this consortium. In anaerobic conditions, nitrate was rapidly removed from wastewater because *B. subtilis* overexpressed nitrogen assimilatory and respiratory genes, significantly enhancing denitrification. They also observed the same phenomenon in a BES using this consortium. The BES effectively removed nitrate by adding *B*. *subtilis*, reaching 96% after 24 h, which greatly impact the microbial community. The population of *Clostridium butyricum* and *C. beijerinckii* increased because of the presence of *B*. *subtilis*, stimulating an increase in the current. Therefore, the BES inoculated with *B*. *subtilis* assimilates ammonium from nitrate-enriched wastewater. In addition to *B. subtilis*, the addition of *G. sulfurreducens* can also promote the degradation rate. Wan et al. [100] constructed the coculture system *G. sulfurreducens* PCA with the denitrifying microbial community. They reported that the interaction between microorganisms eliminated the lag phase of 15 h and improved the denitrification rate by 13%–51%. The mechanism of this enhancement was that *G. sulfurreducens* selectively enhanced the expression of *nirS* coding for a cytochrome cd1-nitrite reductase, resulting in fast and more complete denitrification.

In recent years, the pollution problem caused by dyes has increased considerably, posing a great threat to ecosystems and human health [98]. Nevertheless, conventional technologies (physical and chemical methods) for treating dye pollutants can hardly meet the discharge standard because of the complexity and toxicity of dyes [101]. Generally, DMs can decolorize azo dyes, but the degradation products are frequently toxic metabolites that are even more difficult to degrade. In contrast, microbial consortia could avoid these problems and achieve a higher removal efficiency due to the synergistic metabolic activities [101]. Wang et al. [83] used a consortium composed of two species (*Pseudomonas putida* and *S. oneidensis*) to remove Congo red in a BES. The removal efficiency of the cocultures system reached above 95%, while the Congo red concentration remained unchanged using pure microorganisms over 72 h. In this consortium, *P. putida* functioned more in Congo red degradation and provided more electron donors for *S. oneidensis* by converting Congo red into metabolites. The formation of the biofilm also changed by using this consortium; the area of *P. putida* on the anode surface increased because of the interaction. In addition, dissolved organic matter leached during plastic degradation was a labile carbon-based substrate, which improved the availability of carbon sources and could promote the growth of more microorganisms [102]. This substrate-microbial community interaction relationship is usually complex, and the ubiquitous electron transfer in the microbial community can be determined. However, the detailed description of the power generation performance of microorganisms and the degradation mechanism of microplastics in the system is lacking, and more attention should be paid to the follow-up related research work.

### 5.2 Promotion of EMs pollutant degradation by DMs

The interaction between pure culture microorganisms has been evaluated for the enhanced biodegradation of various organic compounds. DMs may be relatively limited in metabolizing carbon substrates, which can be solved by converting pollutants into simple substrates by EMs. The utilization of mixed cultures can reduce the metabolic burden to facilitate a system to degrade recalcitrant feedstock. Moreover, the range of degradable substrates can be enlarged after adding EMs since the change in metabolic pathways and electrons. Thus, more and more organic compounds and pollutants can be utilized.

Chrysene is a high molecular weight PAH with four fused benzene rings. Due to its refractory properties, the bioavailability of chrysene is very low, and thus persistent in the environment, but can be efficiently degraded by the application of microbial consortia. Vaidya et al. [92] established a consortium consisting of *Rhodococcus* sp., *Bacillus* sp., and *Burkholderia* sp., which could utilize chrysene as a sole source of carbon and energy. The result showed that the maximum degradation by the consortium reached 96%, and chrysene was degraded through the phthalic acid pathway. Glycerol, a refractory substrate, is a major byproduct of the biodiesel conversion process, which is an attractive feedstock because of its mass availability. The use of one single microorganism can hardly degrade glycerol, so the construction of microbial consortia provides an effective choice. Kim et al. [97] used glycerol as the substrate for the coculture of *S. oneidensis* MR-1 and *Klebsiella pneumonia* J2B in an MFC. They reported that the availability of glycerol could reach 97% with simultaneous power production. However, it was not utilized in MFC with single *S. oneidensis* MR-1. Moreover, the consortium could achieve power generation by consuming acidic byproducts (lactate and acetate), whereas they are accumulated in MFC with pure *K. pneumonia*. Therefore, they concluded that the consortium provides a new way for glycerol utilization using the electrode as an electron acceptor. *K. pneumonia* could convert glycerol into other substances that could be used by *S. oneidensis*. The same conclusion was also reported by Kubannek and his group [103]. Cellulose is a macromolecular polysaccharide composed of glucose. Few microorganisms can directly degrade themselves alone but they can degrade their hydrolyzate [13]. Thus, a synergistic consortium of polymer-degrading, fermentative DMs, and fermentation products utilizing EMs would be efficient using cellulose as substrates. Ren et al. [73] used a defined microbial consortium of the cellulolytic microorganisms *Clostridium cellulolyticum* and the EM *G. sulfurreducens* to generate electricity by using cellulose as the substrates in a BES. The removal efficiency of the consortium increased from 42% to 64% compared with the pure *C. cellulolyticum*. Meanwhile, the consortium achieved maximum power densities of 143 mW/m^2^. The thorough utilization of cellulose was because that *C. cellulolyticum* firstly fermented cellulose primarily into acetate, ethanol, and hydrogen as expected, then *G. sulfurreducens* consumed some of these as electron donors.

In a word, the interaction between microorganisms can not only enable the removal of refractory pollutants but also convert the substances that a single microorganism cannot use into energy. Notably, many microorganisms selected to construct the consortia should have the ability to adapt to a specific environment. The ratio of the two microorganisms also needs to be set reasonably. Consequently, these problems need to be further studied and solved.

## 6 Interrelationships between EMs and DMs

As mentioned above, EMs can be divided into exoelectrogens and electrotrophs, both of which play a role in the degradation of organic contaminations, including oxidative degradation on the anode (by exoelectrogens) and reductive degradation on the cathode (by electrotrophs). There is no clear distinction between EMs and DMs. EMs are defined as cells that produce or use extracellular electrons, while degrading bacteria may also possess this characteristic. For example, the electrotrophic microorganisms *Marinobacter* spp. affiliated to *Gammaproteobacteria* are typical hydrocarbon-degrading bacteria [104]. As the first reported pure culture that utilizes extracellular electrons, *Geobacter metallireducens* can reduce nitrate to nitrite on the cathode, and *Alcaligenes faecalis*, *Desulfopila*, *Desulfovibrio*, and *Klebsiella pneumoniae* also perform denitrification under abundant extracellular electrons [104]. The identified DMs usually conduct the key processes of pollutant decomposition, such as the ring opening of aromatic hydrocarbons and the dehalogenation of halogenated hydrocarbons. At the same time, EMs (e.g., *Geobacter*, *Shewanella*, and *Bacillus*) can further utilize small molecule metabolites from pollutants and export extracellular electrons generated by solid electrodes, thereby promoting degradation processes. Notably, the generated biocurrent may also stimulate the growth and activity of EMs. The difference from the communication of information with genetic material to achieve mutual regulation of species is that microorganisms can use hydrogen or formic acid as electron donors to reduce pollutants. This communication on nutrient consumption is thought to make degradation more efficient. Although there is no clear boundary between degrading and EMs, it could be determined that both are important participants in electron transfer of the pollutant degradation pathway. Therefore, we can divide the EMs involved in the metabolism of pollutants into two categories: (1) degrading EMs that participate in the core degradation path of pollutants; and (2) non-degrading EMs that only participate in secondary metabolites such as simple acid salts, alcohols, and esters. In microbial electrochemical systems, the role of exogenous electrodes is to promote the export of extracellular electrons and stimulate the activity of EMs (degrading and non-degrading EMs). That is, BESs can directly or indirectly promote the degradation of contaminated substrates. In addition to the connection between microorganisms and electrodes, the study of electrode ecology formed by electron transfer and interaction between microorganisms share the same important position [105]. Electron transfer mechanisms between microorganisms may also be related to direct cell contact by cytochrome, fimbriae or extracellular appendages, nanowires, and mediators. Microorganisms can synergistically increase power generation capacity, and this synergy can be fully utilized in applications. For example, in cocultivation with exoelectrogens, other bacteria can promote electricity production by removing harmful chemicals or producing current substrates, and even generating a chemical signal to interact with each other [106].

## 7 Conclusions and perspectives

The functionalized microorganisms are fascinating in the development of new biotechnologies such as bioenergy production and pollutant disposals. It has been recognized that social communication among microorganisms greatly enhances the function of single strains. Microbial consortia consisting of various EMs and DMs display a powerful ability for biodegradation of pollutants with the electrical signal output. Although the role of single EMs or DMs has been proven effective through genetic engineering, the best way to break through thermodynamic barriers is to form consortia, which enhances efficiency in bioenergy production and pollutant disposals. For the practical application of consortia between EMs and DMs, some issues need to be considered. First, different microorganisms often possess various growth features, such as temperature, pH, redox, or dissolved oxygen. The social communications between microorganisms must be in growth conditions where microorganisms can survive well to ensure normal life activities and stable coexistence of different species. This can be solved by selecting several microorganisms with similar or complementary living conditions when constructing a microbial consortium. Second, a rational microorganism structure and substrates allocation modes also play vital roles in social communications. Normally, substrate competition (carbon sources and nitrogen sources) exists in microbial consortia, which leads to the uncontrollable growth of each species within microbial consortia. Therefore, more advanced biological methods (for example, phylogenetic and metabolic spectra) are required to further understand interactions and the principle of enhancing bioprocesses to generate bioenergy and degrade pollutants. Third, some mediated materials in the natural environment have been proven to play an essential role in a microbial consortium, enlightening us to popularize their application. Finally, the use of EMs and DMs is based on the premise of solving environmental pollution and the energy crisis. Therefore, the construction and use of microbial consortia should avoid using microorganisms that are harmful to the environment and humans or minimize their harmful effects. All in all, let’s not be surprised by any possible combination of microorganisms. There is always a microbial collocation that will amaze and change the world.

## Author contributions

All authors contributed to the text of the article. Q.Z. wrote the final version and performed all of the necessary editing.

## Declaration of competing interest

The authors declare no conflicts of interest.
